# Low-dose Erythropoietin reduces risk of heart failure induced by anti-cancer therapy

**DOI:** 10.18632/oncotarget.352

**Published:** 2011-11-12

**Authors:** Melanie Hoch, Denise Hilfiker-Kleiner

**Affiliations:** Department of Cardiology and Angiology, Medical School Hannover, 30625 Hannover

Heart disease can be caused by many different insults and injuries and often terminates in death from heart failure, an inability of the heart muscle to adequately supply blood to the body. A major aim in today's research is to define novel therapeutic strategies to limit myocardial damage and to enhance the ability of the heart muscle to regenerate and to heal itself. The anti-cancer drug doxorubicin (DOX) is used to treat a broad range of cancers but is limited in its use by severe side effects, most notably heart failure [1]. STAT3 is an important factor that promotes survival and migration of many tumor cell types and its pharmacological blockade hold good promise as a novel anti-cancer strategy [2, 3]. However, likewise to DOX, blocking STAT3 in the heart has been associated with heart failure in various experimental models [4, 5]. In our latest work, we found that low-doses of erythropoietin (EPO), a cytokine with multiple functions best known for its role to control the production of red blood cells, reduces the risk of heart failure mediated by DOX treatment or STAT3 blockade [6]. Our findings suggest that treatment with low-doses (hematocrit-inactive) synthetic EPO (CERA) during DOX treatment or STAT3 blockade is able to preserve the ability of cardiac progenitor cells to repair the vasculature of the heart and thereby to attenuate myocardial injury [6]. Recent studies have shown that the heart possesses intrinsic cardiac stem or progenitor cells that can contribute to regeneration and healing during disease and aging [7]. Little is known about the molecules and pathways that regulate stem cell proliferation and differentiation. In order to obtain a better understanding how the cardiac microenvironment impacts on the endogenous regeneration system of the adult heart, we studied phenotypic alterations of resident cardiac progenitor cells (identified by the expression of Sca-1) in heart failure as a consequence of DOX treatment or genetic ablation of STAT3 in cardiac muscle cells. We observed that in both heart failure models, cardiac progenitor cells displayed an impaired ability to form new blood vessels essential for oxygen delivery to the heart muscle[6]. Both models also exhibited reduced production of EPO in the heart muscle[6]. Interestingly, we found that cardiac progenitor cells bind EPO within the cardiac microenvironment, a feature that seems to be required to maintain the ability to differentiate into new blood vessels[6]. Importantly, administration of the synthetic EPO derivative CERA at a low-dosage (hematocrit-inactive) restored or preserved the endothelial differentiation potential of cardiac progenitor cells in mice with depleted STAT3 or in mice obtaining DOX treatment. Moreover, CERA preserved heart blood vessels and cardiac function in both mouse models[6]. Our data imply that short-term EPO administration at low-doses seems an attractive avenue to pursue for protecting the heart against chemotherapy-induced failure and might even have broader applications in cardiac regeneration[6]. However, further evaluation of EPO in tumor research is required, especially with regard to its effect on tumor growth and anti-tumor treatment strategies.
